# An Easy and Economical Way to Produce a Three-Dimensional Bone Phantom in a Dog with Antebrachial Deformities

**DOI:** 10.3390/ani10091445

**Published:** 2020-08-19

**Authors:** Hee-Ryung Lee, Gareeballah Osman Adam, Dong Kwon Yang, Tsendsuren Tungalag, Sei-Jin Lee, Jin-Shang Kim, Hyung-Sub Kang, Shang-Jin Kim, Nam Soo Kim

**Affiliations:** 1 College of Veterinary Medicine, Jeonbuk National University, Specialized Campus, Iksan 54596, Korea; hr-lee@hanmail.net (H.-R.L.); gorba000@gmail.com (G.O.A.); dkyang0502@gmail.com (D.K.Y.); mgljuuh@gmail.com (T.T.); kimjs@jbnu.ac.kr (J.-S.K.); kang-hs@jbnu.ac.kr (H.-S.K.); 2 Hansarang Animal Hospital, Seoul 02880, Korea; 3 Department of Veterinary Medicine and Surgery, College of Veterinary Medicine, Sudan University of Science and Technology, P.O. Box 204 Khartoum, Sudan; 4 Korea Basic Science Institute Jeonju Center, Jeonju 54896, Korea; lsj@kbsi.re.kr

**Keywords:** 3-D, antebrachial growth deformities, corrective osteotomy, dog, patient-specific instrumentation, virtual surgical planning

## Abstract

**Simple Summary:**

Accurate planning, for corrective surgeries in case of bone cutting, is necessary to obtain a precise coordination of the skeleton and to achieve the owner’s satisfaction. The present experiment displays a simple and cost-effective technique for surgical planning, utilizing a 3-D bone phantom model in a dog with foreleg deformity.

**Abstract:**

3-D surgical planning for restorative osteotomy is costly and time-consuming because surgeons need to be helped from commercial companies to get 3-D printed bones. However, practitioners can save time and keep the cost to a minimum by utilizing free software and establishing their 3-D printers locally. Surgical planning for the corrective osteotomy of antebrachial growth deformities (AGD) is challenging for several reasons (the nature of the biapical or multiapical conformational abnormalities and lack of a reference value for the specific breed). Pre-operative planning challenges include: a definite description of the position of the center of rotation of angulation (CORA) and proper positioning of the osteotomies applicable to the CORA. In the present study, we demonstrated an accurate and reproducible bone-cutting technique using patient-specific instrumentations (PSI) 3-D technology. The results of the location precision showed that, by using PSIs, the surgeons were able to accurately replicate preoperative resection planning. PSI results also indicate that PSI technology provides a smaller standard deviation than the freehand method. PSI technology performed in the distal radial angular deformity may provide good cutting accuracy. In conclusion, the PSI technology may improve bone-cutting accuracy during corrective osteotomy by providing clinically acceptable margins.

## 1. Introduction

Since the three-dimensional (3-D) fabrication methods developed in 1981 [1,2], this additive manufacturing technology has become possible through the stereo lithography apparatus (SLA) method (in 1984) and fused deposition modeling (FDM) 3-D printing process (in 1988) [3]. In 1992, human body cleft palate was produced using 3-D printing by the SLA method. In 1997, a 3-D printed physical model of a human patient’s joint was used to establish a surgical plan in orthopedic surgery and to improve surgical completion [4]. An unfitted osteotomy can lead to serious iatrogenic translation because of inadequately measured bone deformities [5,6].

In the case of angular limb deformity (ALD) corrective osteotomy, many studies have been conducted using the 3-D technique and RP bone model. In particular, the improvement of the operation time, irradiation time, surgical invasion, surgical accuracy, and patient pain, patient-specific instrumentations’ (PSI) usefulness—such as risk, pre-operative planning, and surgical error reduction—have recently been demonstrated [7,8,9,10,11,12,13,14,15,16,17].

In general, a program that converts computed tomography (CT) data, which is used in many studies, into a 3-D printable file format (stereo-lithography; STL) after bone segmentation and 3-D surgical planning simulation program are expensive and require expertise for operation. Therefore, attempting to request 3-D print production of an affected bone model and patient-specific osteotomy guides from an external company may require more than two weeks’ time, several modifications, and relatively high costs [18,19,20,21]. These drawbacks rendered the application of this technique limited in the surgical field in general, and veterinary field in particular; regardless, the technology has advanced [18,21]. Limited information on surgical-planning techniques are reported in veterinary literature [17,20,22,23,24,25,26,27]. In this study, we employ the PSI for corrective osteotomy using a rapid prototyping (RP) technique. Our technique utilizes in-home facilities, such as 3D-printers and free software packages, that are affordable to many practitioners.

The PSI can be applied to joint replacement, joint resurfacing for artificial cartilage implantation, and corrective osteotomy cutting and drilling guides [28,29,30]. The purpose of this study was to compare the results of the PSI-assisted osteotomy with the freehand osteotomy performed on the three-dimensionally fabricated ghost radial bone models and to evaluate the reproducibility and accuracy of each technique. Also, an in-progress protocol with low cost and simplified process was demonstrated.

## 2. Materials and Methods 

All animal procedures were conducted in accordance with the Guide for the Care and Use of Laboratory Animals published by the US National Institute of Health (NIH Publication no. 85-23, revised 1996). The study protocol was reviewed and approved by the Committee on the Care of Laboratory Animal Resources, Jeonbuk National University with an ethical approval number of CBNU2018-0044. The study was conducted at Hansarang Animal Hospital (Seoul, Republic of Korea) and College of Veterinary Medicine, Jeonbuk National University (Iksan, Republic of Korea).

To improve accessibility in the veterinary orthopedic field, low-cost products were selected for less than $500 (FDM 3-D printer; $350, PLA filaments; $15, open source free software, etc.). 

### 2.1. Case Presentation and Imaging

**Figure 1:**
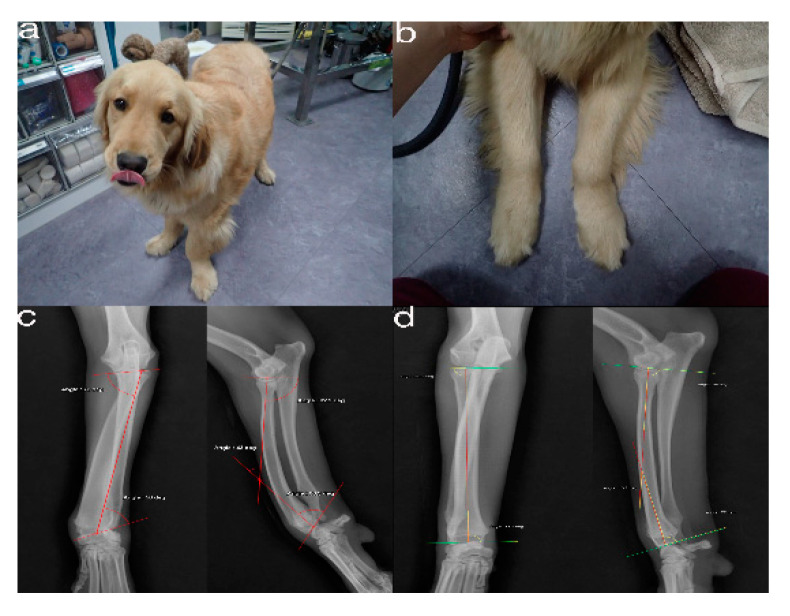
The images show the antebrachial growth deformity (AGD)-affected (left leg) and contralateral unaffected-antebrachium (right leg) with the joint-orientation angles (**a**,**b**), and orthogonal radiographs (**c**,**d**).

**Figure 2:**
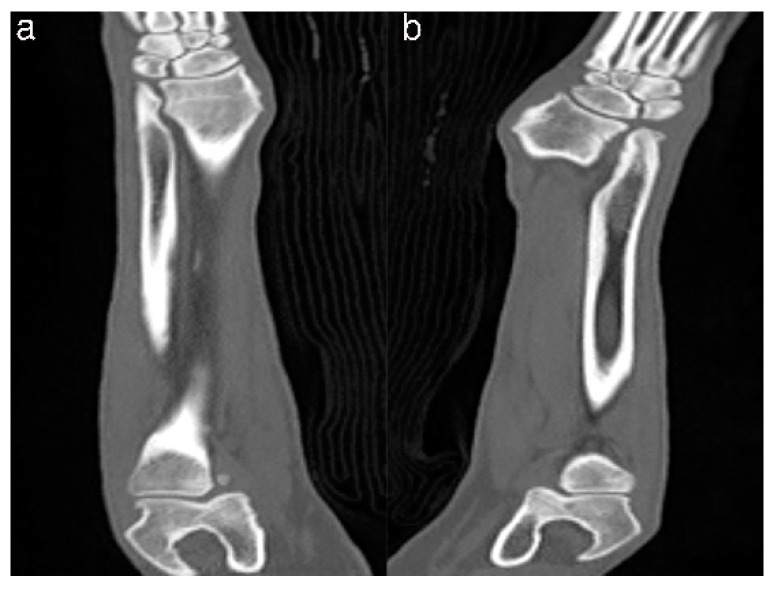
CT from the distal third of the humerus to the metacarpal bones of both the affected (**a**) and unaffected leg (**b**).

**Figure 3:**
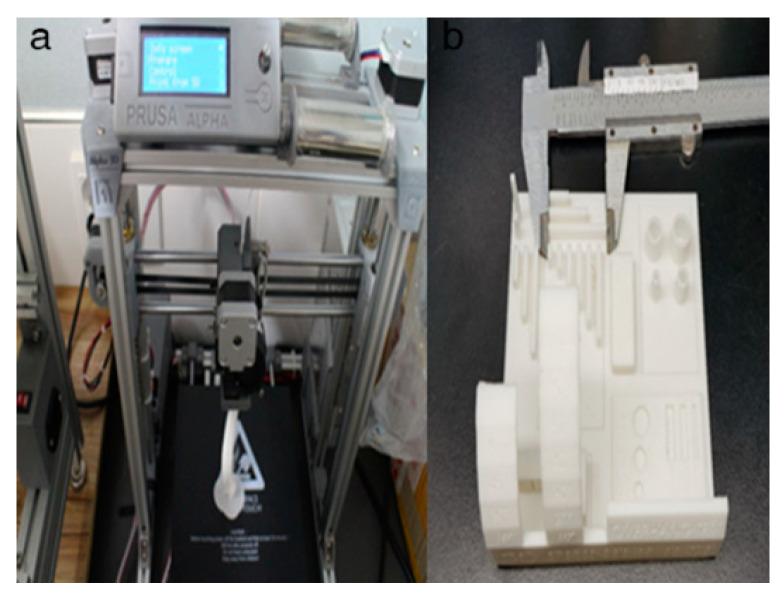
Printer for the in-house fabrication. In this study, the desk-top fused deposition modeling 3-D printer was employed for the in-house fabrication from polylactic acid filaments (**a**). Before the thesis research work, accuracy checking process for this 3-D printer was performed using 3-D accuracy-test tool (**b**).

**Figure 4:**
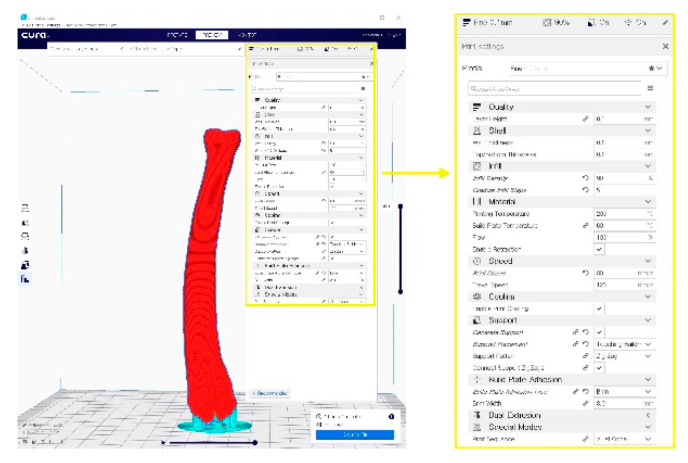
A G-code file generating process for the 3-D printing process. It can be generated from the STL image using the CURA application program.

The simulated bones consisted of the forelimb angular deformity model in a 14-month-old, female Golden Retriever dog weighing 28.4 kg presented with less weight-bearing on the lower left forelimb, restricted flexion and pronation/supination, radial shortening due to premature closure of the epiphyseal growth plates, and pain due to kinetic chain dysfunction (Figure 1a,b). Orthogonal radiographs were taken to evaluate the orientation and the site of the maximal radio-ulnar angular deformity (Figure 1c,d). CT images (slice thickness 0.7 mm; 120 kV; helical CT Alexion, Toshiba, Japan) were obtained from the distal third of the humerus to the metacarpal bones of both the affected and unaffected legs (Figure 2). The affected and unaffected side structures of the radius and delineated from CT images of a patient with angular deformity who scheduled for corrective osteotomy. 3-D slicer freeware (3-D Slicer, https://www.slicer.org/; version 4.8.0) was used to convert bone surface image information into a SLT file format. Preoperative planning of the osteotomy was presented by a CAD program (3-D Builder; Microsoft windows application, www.microsoft.com) with the SLT images of the distal radius. The CAD file of the cutting guide renders virtually placed in the affected area, and cutting planes placed on the virtual 3-D bone models (Figure 2). The previously extracted SLT models from the antebrachia were used in 3-D printers by using special g-codes that were generated by a free software (CURA; https://ultimaker.com/software/ultimaker-cura) (Figure 3). The experiments were organized using patient-specific phantom models fabricated in-house from the PLA filaments using FDM 3-D printing (Alpha-i3, Alpha3-D, Korea) (Figure 4).

### 2.2. Virtual Corrective Osteotomy

**Figure 5:**
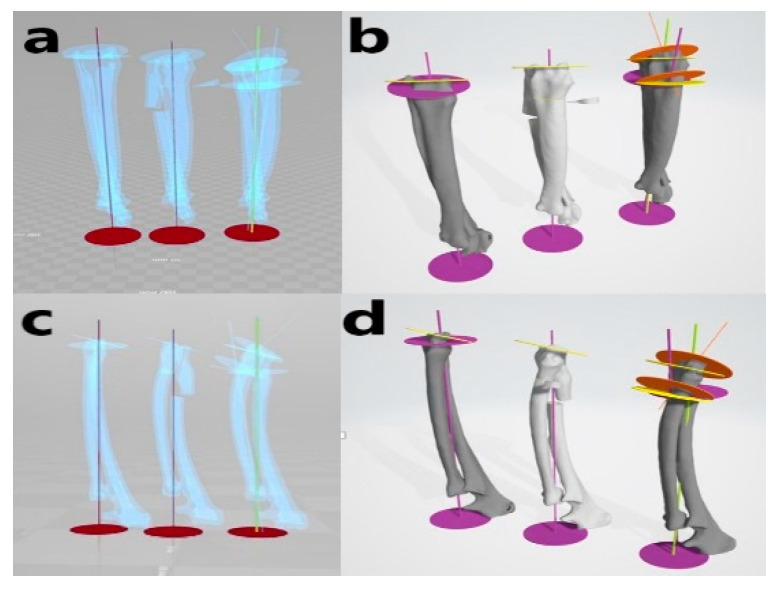
Virtual simulative corrective osteotomy. Surgical simulations were performed in virtual reality to predict postoperative conditions. Note the change in the axis before and after the virtual corrective osteotomy. The figure on the left (**a**,**c**) shows penetrated images and on the right (**b**,**d**) are 3-D rendering images.

Virtual osteotomy was performed (Figure 5) along the osteotomy plane designed previously, followed by correction of the deformity using the ‘activate’ or ‘deactivate’ object mode to rotate and move functions using the 3-D builder (Microsoft windows free application program, Microsoft Corporation, Washington, DC, USA). The affected radius model was cut into segments and the distal bone segment was repositioned. The plates, PL1”-PL2’ were designed and appropriate osteotomy planes at the optimum cutting points (TJ-PL2’) were selected, followed by choosing the ‘subtract’ function in the edit mode for plates on the osteotomy plane that intersected the affected radial model. The correction was confirmed by measuring appropriate angles. For the collinear realignment, we virtualized the opening wedge, closing wedge, neutral wedge, and dome osteotomy, then confirmed our surgical planning based on the concepts such as the CORA, multiple osteotomies, anatomical and mechanical axes, angulation correction axis, and the bisector. We also considered patient factors and fixation methods to determine the surgical plan.

### 2.3. PSI Design for the Distal Radial Surface

**Figure 6:**
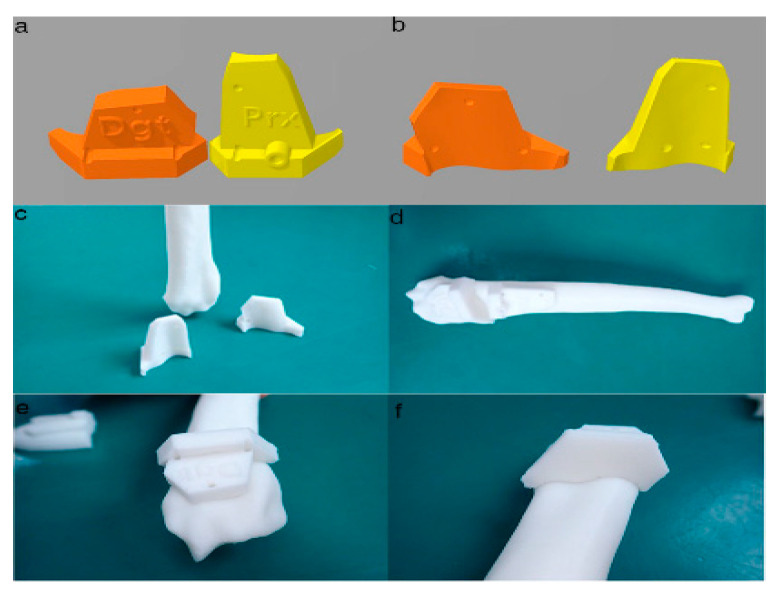
Final designs of patient-specific instrumentation (PSI) models were demonstrated through 3-D rendering images (**a**,**b**), 3-D printed PSIs (**c**), and the PSIs positioning on a rapid prototyping (RP) bone model precisely (**d**–**f**). The patient-specific cutting guides added a drill guide for the bone plate and screw placement. The PSIs attached on the RP bone model in-situ (**d–f**).

Two PSIs designed for each plane cuts on the radius. Each PSI was created to fit the unique surface prominences and depressions of the osteotomy site of the bone surface and establishes a flat surface representing the target cutting plane (Figure 6). Each PSI was designed with guide holes for 1.2-mm K-wires that were used for temporarily fixing the PSI on the bone during osteotomy and could also be used as predrilling holes for plate and screw fixation. After the PSI design was approved, PSIs were manufactured in-house using RP technology with a desktop fused filament fabrication 3-D printer (Alpha-i3, Alpha3-D, Seoul, Korea) in a PLA material.

### 2.4. In-House Fabrication

**Figure 7:**
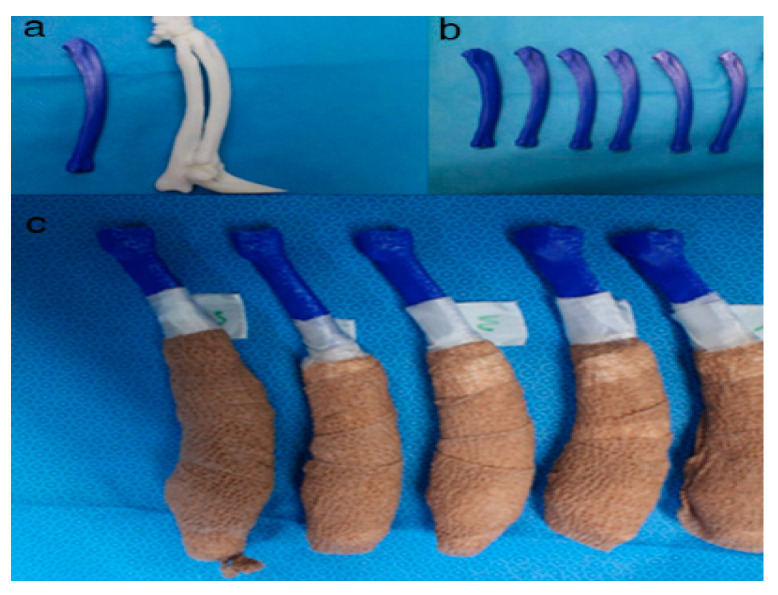
Rapid prototyping bone models were fabricated with the polylactic acid material using the fused deposition modeling method (above, **a**,**b**) and then the phantom bone models were fabricated for this study (below, **c**).

Ten real-sized RP bone models were manufactured in-house using the FDM 3-D printer and a phantom bone model was fabricated. RP bone models were wrapped by cotton rolls followed by self-adhesive elastic tapes (Coban, 3M, St. Paul, MN, USA) (Figure 7).

The PSIs were provided with 1.2 mm-diameter pin guides for temporary fastening to the radial bones using K-wires or bone forceps. The needles were used during surgery to find the PSI guidance location as set in the preoperative plan.

### 2.5. Rehearsal Surgery on the Phantom Bone Models with PSI

**Figure 8:**
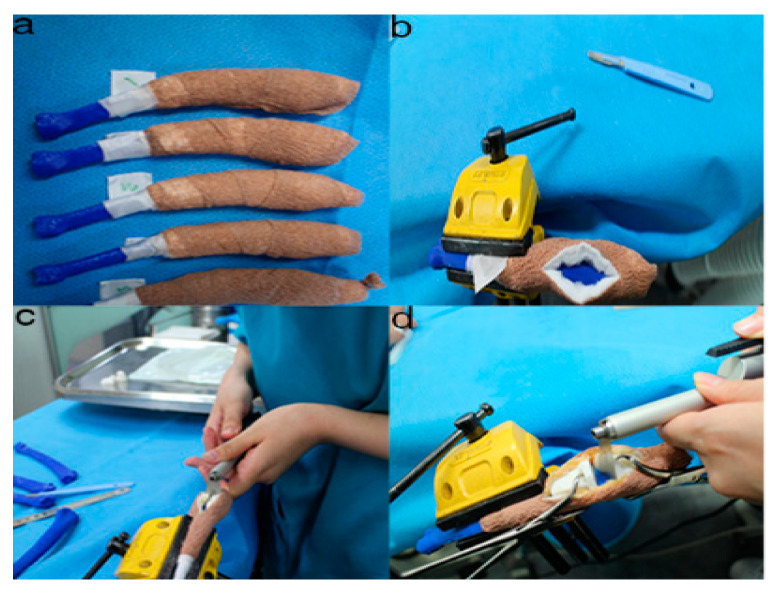
The position and angles for the cutting procedures. Prior to the ex-vivo osteotomy, each of the five operators were directed to position the phantom bone model to a vise (**a**,**b**). The surgical planning conditions for the freehand method were the same as the patient-specific instrumentation (PSI) method (**c**). Positioning PSI was ordered without positioning aids, but was only fixed on the rapid prototyping model using the bone-holding forceps or K-wires for temporary fixation (**d**).

**Figure 9:**
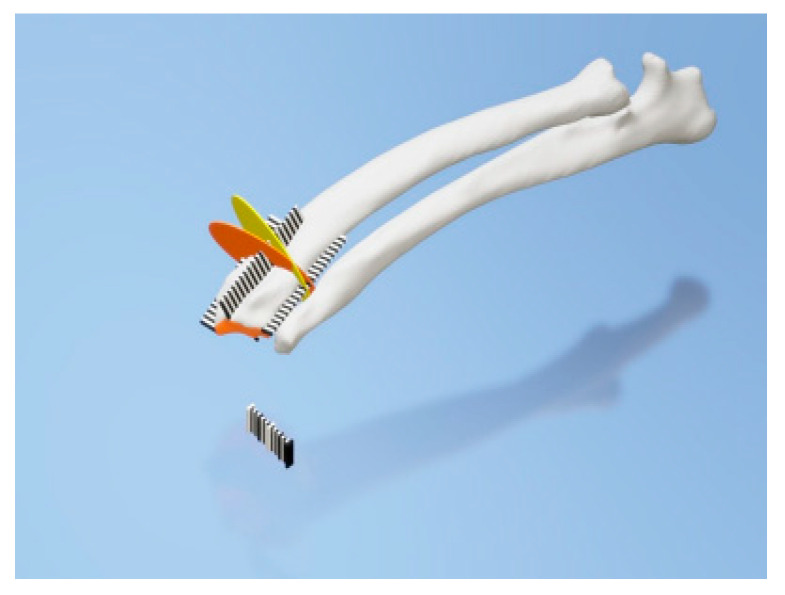
Rulers were attached to the cranial, caudal, medial, and lateral bone model surfaces. The orange and yellow plates show the osteotomy target plane. The black and white striped bar is made 1 mm against each space to indicate the distance from the edge of the joint surface.

Five operators individually performed two cuts using PSI and freehand on the phantom bone models. Before sawing, each operator was directed to position the two PSIs without an instrument and fix them on the phantom radial bone model using the bone holding forceps or K-wires to prevent movement. After sawing, each operator was directed to take off the bone holding forceps. The bone model was fixed using a steel vise (Multi-Angle Base Vise 83-069, with a multi-angle rotating ball joint system) to a proper position for the simulation (Figure 8). To avoid errors while sawing, the order was given to the target plane as precisely as possible. For the freehand cutting, a craniomedial approach to the distal radius model was completed. An osteotomy was performed by an oscillating saw 25 mm apart from the carpal joint margin in parallel (Figure 9). A second osteotomy was ordered at a fixed angle of 25 degrees relative to the first osteotomy at the corrective angle. The positions and angles for the cutting planes were the same as the surgical planning conditions given in the PSI designed on the CAD software. Before freehand cutting, each operator was asked to decide and place k-wires as a guidance navigator, and the sawing was done by hand with an oscillating saw without a cutting guide. The total time for the bone cutting was defined by the time spent from the point of exposing the bone-cutting site until the end of bone cutting. In the freehand group, the total time included the arrangement time of placement of the navigation K-wire and intraoperative control of the correct osteotomy angle under ruler measurement. In the PSI group, the total time included positioning and temporarily fixing the PSI on the model surface and then finish the sawing under PSI guidance. 

### 2.6. Accuracy Evaluation

PSI technology was used to evaluate 20 intersection data sets for a precise cut using two parameters. The accurate location and angulation were used to evaluate the geometrical accuracy of the cut planes. The bone phantom model and PSI technology were performed to assess the effect of the variables between distal and proximal bone cut planes and among a group of operators. The variable ‘operator’ was considered as a random effect. The angle of cutting planes and positional accuracy were considered two numerical responses of the model. Statistical analysis was performed by using Fisher’s exact test and the statistical differences for both the location and angle using were investigated their logarithms to base 10. The *p*-value of less than 0.05 was considered statistically significant. The post-hoc Tukey’s tests were conducted to determine whether the target variable was different in terms of response variables and if the effect of the variable ‘target plane’ was significant (*p* < 0.05).

## 3. Results

The images revealed abnormal lateral orientation at the distal part of the radius and ulna, proximal to the carpal joint. Craniocaudal and mediolateral radiographic projections showed a multiplane antebrachial bone deformity (Figure 1c,d).

**Figure 10:**
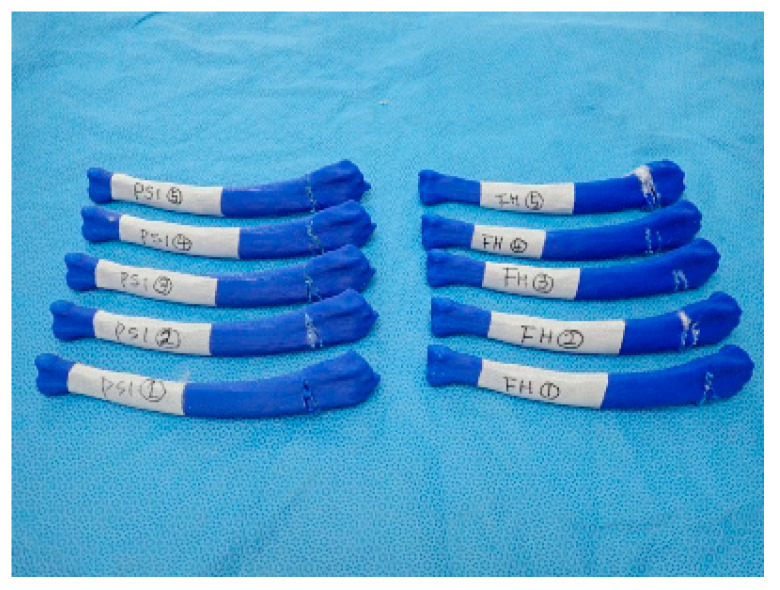
Simulative osteotomy result on phantom bone models. Patient-specific instrumentation (PSI) and freehand (FH) marked rapid prototyping bone models are the result of the osteotomy using the PSI and freehand, respectively. This picture was taken after the ex-vivo osteotomies were performed on the phantom bone models and the outer materials were removed.

Twenty cut planes (4 out of the 5 operators) were used for estimation of the cutting performance with PSI procedure (Figure 10). The proper position of the cut planes and the obtained surgical margins were subjected to notable differences regarding the average and 95% confidence interval (CI) between the four target planes. The location precision of the PSI-assisted cutting distance from the target on the medial and lateral radius (mean 0.2 mm, 95% CI [−0.1016, 0.5016] and 0.2 mm, 95% CI [−0.1016, 0.5016], respectively; both *p*-values < 0.0001) were significantly different from that of the freehand technique (average 4.1 mm, 95% CI [2.774, 5.426], *p* = 0.0270 and 4.4 mm 95% CI [2.516, 6.284], *p* > 0.10, respectively). The freehand marginal point achieved in the lateral (average 4.4 mm, 95% CI [2.516, 6.284]) were significantly higher than that achieved in the PSI (average 0.2 mm, 95% CI [−0.1016, 0.5016], *p* < 0.0001), as shown in Table 1. 

**Figure 11:**
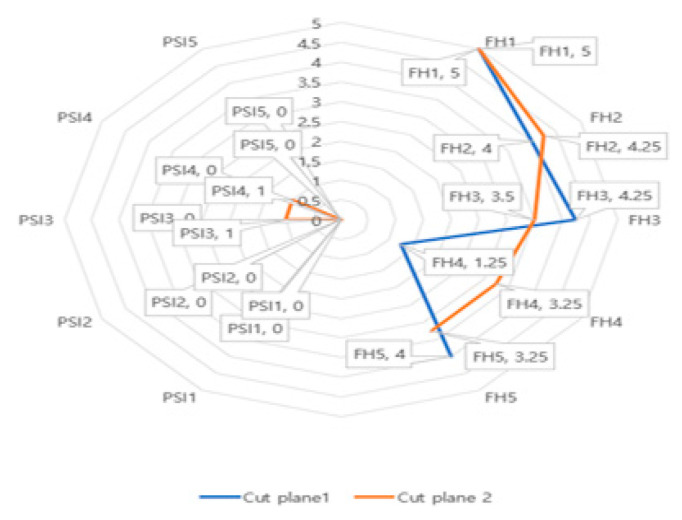
Distance (mm) from the target plane to the cut plane: freehand (FH) versus patient specific instrument (PSI). The numbers on the center to the 12 o’clock direction are the indicated distances in millimeters from the cutting target position. The PSI group (left) and the FH group (right) side were placed. The results are shown separately by dividing the cut-planes 1 and 2 in each text box.

The maximum distance between the achieved marginal point and the planned marginal point was found in the lateral area of the marginal point (3 and 0 mm for junior and senior surgeons, respectively). The 2-mm desired safe margin is showed by the solid line ‘target’ on Figure 11, (a) *p* < 0.05 compared with freehand; and (b) *p* < 0.05 compared with PSI. The angulation of the cut planes was subjected to insignificant variations between the target planes. The cutting angle error from the target cutting angle between the distal and proximal cut planes in the freehand group (mean 8.558 mm, 95% CI [−18.33, 1.210]) was faintly distinct from that of the PSI group (mean −0.004 mm, 95% CI [0.032, 0.014]).

**Figure 12:**
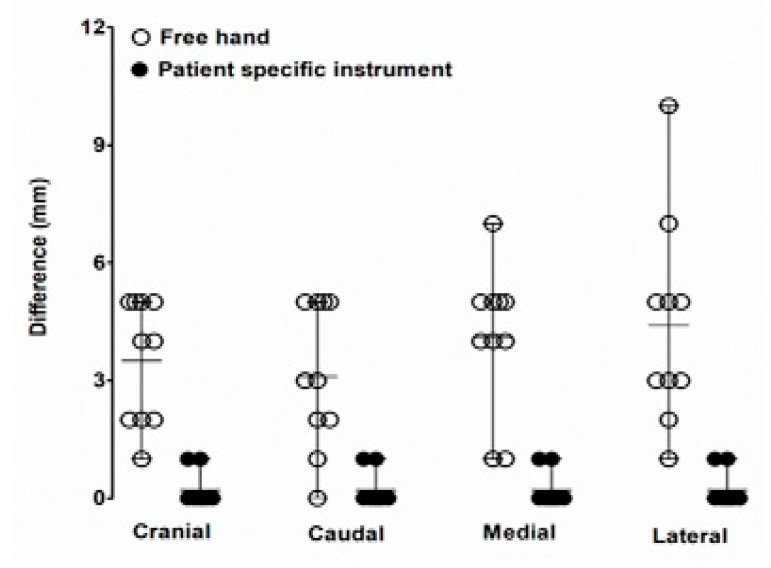
Distance (mm) from the target plane to the cut plane: freehand versus patient-specific instrumentation (PSI). The measurement point was set on the cranial, caudal, medial, and lateral surfaces of the rapid prototyping phantom bone model. In the PSI group, accuracy test results showed less than 1 mm distance from the target plane. On the other hand, the freehand method group results showed up to 11 mm of error from the target plane.

**Figure 13:**
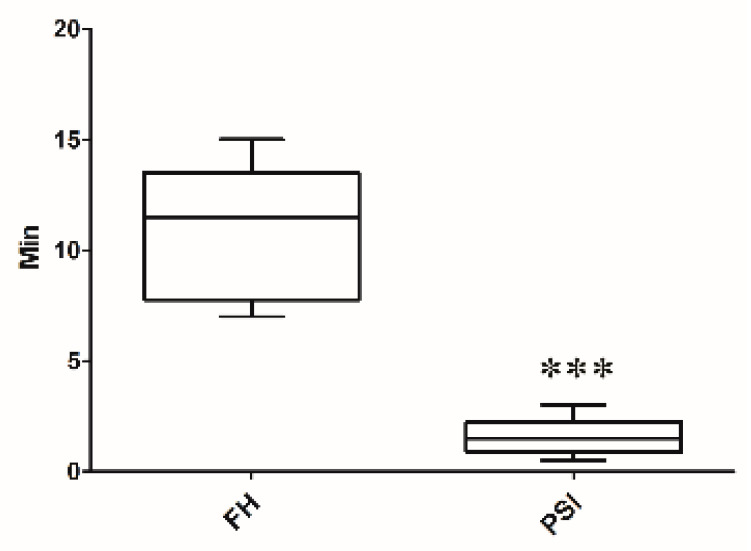
Boxplot displaying the time consumption for the cutting procedure after applying the two different methods: freehand (FH) versus patient-specific instrumentation (PSI). *** *p* < 0.001, *t*-test *vs.* FH.

**Figure 14:**
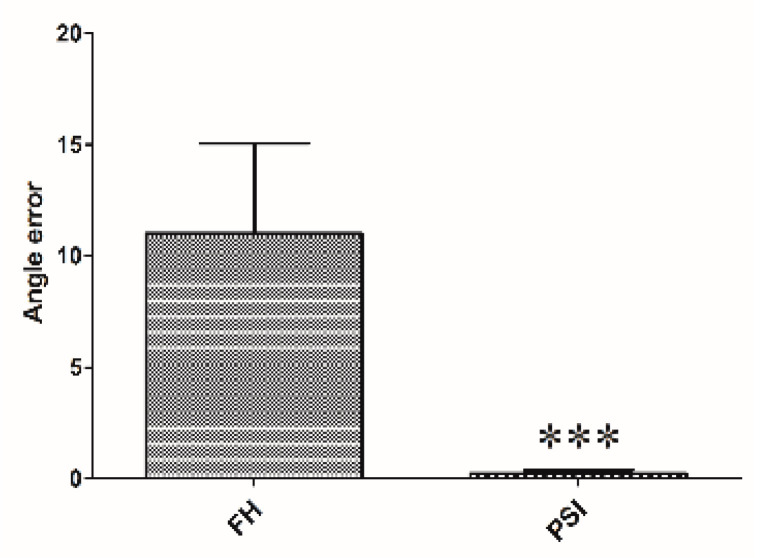
Boxplot displaying the correction angle error of the cutting procedure after applying the two different methods: freehand (FH) versus patient-specific instrumentation (PSI). *** *p* < 0.001, *t*-test *vs.* FH.

The mean time consumed for cutting to complete two planes in each phantom model was 7.9 ± 4.8 min and 1.6 ± 1.1 min for the freehand and PSI, respectively (Figure 12). Measurement point was set on the cranial, caudal, medial, and lateral surfaces of the rapid prototyping phantom bone model. In the PSI group, accuracy test results showed less than 1 mm distance from the target plane. On the other hand, the freehand method group results showed up to 11 mm of error from the target plane. The PSI manner required significantly less time than the freehand models (*p* < 0.0001). The operating time was 6.9 min (range, 5.6–7.9 min) in the senior surgeons and 8.1 min (range, 5.3–12.4 min) in the junior surgeons (Figure 13), in which the operation time consumption of the FH group appears higher than the PSI user group. The box plot of angular errors is shown in Figure 14, where the correction angle error score of the FH group appears higher than the PSI user group.

## 4. Discussion

Since over- and under-correction could lead to the loss of restoration of range motion, the accuracy of pre- and post-operative reconstruction is critical [31]. Hence, computerized 3-D comparisons of the contralateral bones are useful for preoperative planning of the corrective osteotomy in the complicated angular deformities and complex malunions. Some technologies, such as navigation systems and PSIs, have assisted surgeons to perform as accurate as planned in human orthopedic medicine [32,33]. In particular, PSIs are becoming more popular because they are easier to handle than navigation systems and are more precise than free hand in vitro study [34].

To compare the traditional CORA measurement orthogonal radiographs with a CT-defined wedge, we utilized radiographs of both models. Fixation of elbow of the model rendered replication of the dog’s limb difficult. Regardless, two methods of measurement yielded significantly different results with terms to the wedge angle and orientation. The use of 3-D CT reconstruction, to identify the true orientation of joint surfaces to apply the CORA approach, has not been previously described in animals. 

Meola et al. performed CT scans to assess radial torsion in the presence of procurvatum and valgus deformity in dogs [35]. CT data are being extensively used in human studies [36]. To provide guidance for correction, unaffected opposite 3-D CT data should be collected to serve as reference data. In orthopedic surgery, a similar technique to our method has been described, but instead of estimating a normal for the curvature of the bone from the joint surface, the contralateral limb was used as a guide [37]. However, one of the drawbacks of this technique is that the contralateral limb is also affected by deformity, this method may fail to replicate the normal anatomy. 

Fox et al. have described the mean medial proximal radial angle the mean lateral distal radial angle as 85° and of 87°, respectively, in the frontal plane [27]. 4.88° was the calculated mean torsion in the transverse plane in three dogs [35]. However, these data were based on radiographs calculations and have not been determined from CT reconstructions. Additionally, these data were obtained from medium-to-large, non-chondrodystrophic dogs, and as observed, these angles are likely to vary among breed [27]. Studies using 3-D CT to determine the lateral and cranio-caudal planes accurately and the degree of torsion are useful to guide the future computer-assisted surgeries for ALD. 

In one study that applied a two-level corrective osteotomy, just 44% of surgical cases were adjusted in both the frontal and sagittal planes [22]. Therefore, the authors pursued a one-level amendment in this case. To perform a single closing wedge osteotomy accurately, we brought a 3-D CT scan, virtual rehearsal surgery with 3-D Builder, a free software into surgical planning protocol. According to our knowledge, the use of this free 3-D design application to simulate a proposed surgery has not been previously published in veterinary surgery. This data was subsequently used to generate a stereolithographic image in plastic which could be handled by the surgeon in rehearsal surgeries and in the operating room. This guaranteed the accuracy of the intra-operative osteotomy as the fragments were aligned to the same angulation as the surgical plan that allowed fixation of plates to be performed precisely.

The study attempted a surgical protocol that was simple to deploy in veterinary surgery and could be run at a low cost. That is why we had to plan a wedge technology with a straight oscillating saw blade rather than choosing to use a radial or domed saw blade. This dog’s highly dynamic character would require an additional orthogonal directional bone plate placement.

This study showed good precision while using the PSI procedure during simulated osteotomy of the radial bone angular deformity. Osteotomy angulation in the distal and proximal plane assessed in articles of the ISO1101 location parameter indicated how PSI is reliable in the operating room to replicate a preoperative cutting plan on a distal radius with clinical accuracy [38].

Our results agree with those of Oka et al. [39], which found average residual errors of simulated osteotomy using patient-specific guides of less than 1° and 1 mm. Investigations using PSI technology had already described promising clinical outcomes. However, their evaluation was only based on intraoperative plane images using image intensifiers. Moreover, the amount of correction and the demographic data of the patients were not exposed.

The presented results revealed that the PSI cutting with regard to the location and surgical angles is quite precise. The data presented in this study could be beneficial for the quantitative comparison of various PSI design processes for bone-cutting within the end of the long bone. The presented results did not suggest any notable difference between experienced and inexperienced surgeons using PSI technology with regard to the location accuracy and cutting angles achieved. It appears that the PSI technology may be ready to use by experienced as well as inexperienced digital-aged surgeons.

Results with regard to the location accuracy showed that PSIs enabled surgeons to repeat preoperative cutting planning with good accuracy (Figure 3, Figure 4 and Figure 5). Otherwise, the value added by the PSI technology in improving bone-cutting accuracy was similar to that of the planned osteotomy target points and planes. The results regarding the obtained surgical margins (Figure 3, Figure 4, Figure 5 and Figure 6) suggest that PSI technology tended to provide a smaller standard deviation than freehand. Otherwise, the manual cutting errors were minimum with the PSI technology compared to those with the freehand method. Further research should be carried out to verify the results observed in this study.

The operation time measured was about 4.8 min bone cutting time during the phantom bone surgery using PSI, similar to the cadaveric long bone resection surgery by Sarah et al. [40]; moreover, compared to the previous report [40]. This study confirmed that PSI technology save time for surgeons compared with other techniques—i.e., in less than 10 min with PSI versus up to 16 min in their study. This can be interpreted by the predefined optimal location of the PSI on the bone surface, which serves as an intuitive and natural transfer of the surgical plan in comparison with the time-consuming manual image-to-patient enrollment phase of the procedure (Figure 3, Figure 4, Figure 5, Figure 6, Figure 7 and Figure 8). Given the time-consuming factors—such as the presence of muscles and nerves, bleeding, patient movements, etc.—further in vivo research will be performed to verify the results observed here.

## 5. Conclusions

It was demonstrated that using PSI for simulated 3-D bone cuts of the distal radial angular deformity provides an accurate cutting. This study also evaluated PSI technologies quantitatively in their proper location and obtained surgical margins during simulated bone cuts of the deformed radii. In-vivo, ergonomic studies may be performed here in terms of accuracy, reproducibility, and time-consumption. In total, the PSI technology may improve bone-cutting accuracy during corrective osteotomy by offering clinically acceptable margin. Accordingly, practitioners and clinicians can benefit of this cheap and accurate technology to build a 3-D simulated preoperative bones for surgical planning which can be done inside the clinic without outside assistance. 

## Figures and Tables

**Table 1:** Distance (mm) from the target plane to the cut plane: freehand (FH) vs. patient-specific instrumentations (PSI).

	Cranial 1	Caudal 1	Medial 1	Lateral 1	Cranial 2	Caudal 2	Medial 2	Lateral 2
Target	0	0	0	0	0	0	0	0
FH 1	5	5	5	5	5	5	5	5
FH 2	2	3	1	10	4	2	4	7
FH 3	5	2	7	3	4	1	4	5
FH 4	1	0	1	3	2	5	4	2
FH 5	5	5	5	1	2	3	5	3
PSI 1	0	0	0	0	0	0	0	0
PSI 2	0	0	0	0	0	0	0	0
PSI 3	0	0	0	0	1	1	1	1
PSI 4	0	0	0	0	1	1	1	1
PSI 5	0	0	0	0	0	0	0	0
*t*-test FH vs. PSI	0.015	0.034	0.034	0.034	0.046	0.022	0.000	0.008

## References

[1] Kodama H.A. (1981). Scheme for three-dimensional display by automatic fabrication of three-dimensional model. IEICE Trans. Electron..

[2] Kodama H. (1981). Automatic method for fabricating a three-dimensional plastic model with photo-hardening polymer. Rev. Sci. Instrum..

[3] Adepu S., Dhiman N., Laha A., Sharma C.S., Ramakrishna S., Khandelwal M. (2017). Three-dimensional bioprinting for bone tissue regeneration. Curr. Opin. Biomed. Eng..

[4] Goto M., Katsuki T., Noguchi N., Hino N. (1997). Surgical simulation for reconstruction of mandibular bone defects using photocurable plastic skull models: Report of three cases. J. Oral Maxillofac. Surg..

[5] Iolascon G., Gimigliano F., Moretti A., De Sire A., Migliore A., Brandi M.L., Piscitelli P. (2017). Early osteoarthritis: How to define, diagnose, and manage. A systematic review. Eur. Geriatr. Med..

[6] Escott B.G., Kelley S.P. (2012). Management of traumatic physeal growth arrest. Orthop. Trauma.

[7] Crosse K.R., Worth A.J. (2010). Computer-assisted surgical correction of an antebrachial deformity in a dog. Vet. Comput. Orthop. Traumatol..

[8] Knapp J.L., Tomlinson J.L., Fox D.B. (2016). Classification of angular limb deformities affecting the canine radius and ulna using the center of rotation of angulation method. Vet. Surg..

[9] Savio G., Baroni T., Concheri G., Baroni E., Meneghello R., Longo F., Isola M. (2016). Computation of femoral canine morphometric parameters in three-dimensional geometrical models: 3d morphometric parameters in canine femur. Vet. Surg..

[10] Murphy S.B., Kijewski P.K., Simon S.R., Chandler H.P., Griffin P.P., Reilly D.T., Penenberg B.L., Landy M.M. (1986). Computer-aided simulation, analysis, and design in orthopedic surgery. Orthop. Clin. N. Am..

[11] Sangeorzan B.J., Sangeorzan B.P., Hansen S.T., Judd R.P. (1989). Mathematically directed single-cut osteotomy for correction of tibial malunion. J. Orthop. Trauma.

[12] Vaishya R., Patralekh M.K., Vaish A., Agarwal A.K., Vijay V. (2018). Publication trends and knowledge mapping in 3D printing in orthopaedics. J. Clin. Orthop. Trauma.

[13] Ippolito R., Iuliano L., Gatto A. (1995). Benchmarking of rapid prototyping techniques in terms of dimensional accuracy and surface finish. CIRP Ann..

[14] Webb P.A. (2000). A review of rapid prototyping (RP) techniques in the medical and biomedical sector. J. Med. Eng. Technol..

[15] Meyer D.C., Siebenrock K.A., Schiele B., Gerber C. (2005). A new methodology for the planning of single-cut corrective osteotomies of mal-aligned long bones. Clin. Biomech. Bristol Avon.

[16] Pettitt R., Fox R., Comerford E., Newitt A. (2012). Bilateral angular carpal deformity in a dog with craniomandibular osteopathy. Vet. Comput. Orthop. Traumatol..

[17] Arzi B., Cissell D.D., Pollard R.E., Verstraete F.J.M. (2015). Regenerative approach to bilateral rostral mandibular reconstruction in a case series of dogs. Front. Vet. Sci..

[18] Hespel A.M., Wilhite R., Hudson J. (2014). Invited review—Applications for 3D printers in veterinary medicine. Vet. Radiol. Ultrasound.

[19] Castilho M., Rodrigues J., Vorndran E., Gbureck U., Quental C., Folgado J., Fernandes P.R. (2017). Computational design and fabrication of a novel bioresorbable cage for tibial tuberosity advancement application. J. Mech. Behav. Biomed. Mater..

[20] European Society of Articial Organs (2019). Proceedings of the 46th ESAO Congress, Hannover, Germany, 3–7 September 2019: Abstracts. Int. J. Artif. Organs.

[21] Singhal A.J., Shetty V., Bhagavan K.R., Ragothaman A., Koneru G., Agarwala M. (2016). Improved surgery planning using 3-D printing: A case study. Indian J. Surg..

[22] Worth A.J., Crosse K.R., Kersley A. (2019). Computer-Assisted Surgery Using 3D Printed Saw Guides for Acute Correction of Antebrachial Angular Limb Deformities in Dogs. Vet. Comput. Orthop. Traumatol..

[23] Longo F., Penelas A., Gutbrod A., Pozzi A. (2019). Three-dimensional computer-assisted corrective osteotomy with a patient-specific surgical guide for an antebrachial limb deformity in two dogs. Schweiz. Arch. Tierheilkd..

[24] João B., Dias M.I., Luís C., Requicha J.F., Viegas C.A.A., Jean B. (2017). A 3D printed model for radius curvus surgical treatment planning in a dog. Pesqui. Vet. Bras..

[25] Dorbandt D.M., Joslyn S.K., Hamor R.E. (2017). Three-dimensional printing of orbital and peri-orbital masses in three dogs and its potential applications in veterinary ophthalmology. Vet. Ophthalmol..

[26] Jeong B., Jung J., Park J., Jeong S.M., Lee H. (2016). 3D-printing bone model for surgical planning of corrective osteotomy for treatment of medial patellar luxation in a dog. J. Vet. Clin..

[27] Fox D.B., Tomlinson J.L., Cook J.L., Breshears L.M. (2006). Principles of uniapical and biapical radial deformity correction using dome osteotomies and the center of rotation of angulation methodology in dogs. Vet. Surg..

[28] Rosseels W., Herteleer M., Sermon A., Nijs S., Hoekstra H. (2019). Corrective osteotomies using patient-specific 3D-printed guides: A critical appraisal. Eur. J. Trauma Emerg. Surg..

[29] Wong K.C. (2016). 3D-printed patient-specific applications in orthopedics. Orthop. Res. Rev..

[30] Popescu D., Laptoiu D. (2016). Rapid prototyping for patient-specific surgical orthopaedics guides: A systematic literature review. Proc. Inst. Mech. Eng. H.

[31] Schemitsch E., Richards R. (1992). The effect of malunion on functional outcome after plate fixation of. J. Bone Jt. Surg. Am..

[32] Athwal G.S., Ellis R.E., Small C.F., Pichora D.R. (2003). Computer-assisted distal radius osteotomy. J. Hand Surg..

[33] Croitoru H., Ellis R., Prihar R., Small C., Pichora D. (2001). Fixation-based surgery: A new technique for distal radius osteotomy. Comput. Aided Surg..

[34] Ma B., Kunz M., Gammon B., Ellis R.E., Pichora D.R. (2014). A laboratory comparison of computer navigation and individualized guides for distal radius osteotomy. Int. J. Comput. Assist. Radiol. Surg..

[35] Meola S.D., Wheeler J.L., Rist C.L. (2008). Validation of a technique to assess radial torsion in the presence of procurvatum and valgus deformity using computed tomography: A cadaveric study. Vet. Surg..

[36] Bindra R.R., Cole R.J., Yamaguchi K., Evanoff B.A., Pilgram T.K., Gilula L.A., Gelberman R.H. (1997). Quantification of the radial torsion angle with computerized tomography in cadaver specimens. J. Bone Jt. Surg. Am..

[37] Mahaisavariya B., Sitthiseripratip K., Oris P., Tongdee T. (2006). Rapid prototyping model for surgical planning of corrective osteotomy for cubitus varus: Report of two cases. Inj. Extra.

[38] Cartiaux O., Paul L., Docquier P.L., Francq B.G., Raucent B., Dombre E., Banse X. (2009). Accuracy in planar cutting of bones: An ISO-based evaluation. Int. J. Med. Robot..

[39] Oka K., Murase T., Moritomo H., Goto A., Nakao R., Yoshikawa H., Sugamoto K. (2011). Accuracy of corrective osteotomy using a custom-designed device based on a novel computer simulation system. J. Orthop. Sci..

[40] Bosma S.E., Wong K.C., Paul L., Gerbers J.G., Jutte P.C. (2018). A cadaveric comparative study on the surgical accuracy of freehand, computer navigation, and patient-specific instruments in joint-preserving bone tumor resections. Sarcoma.

